# Supplementary material for: Identification of photoperiod-induced specific miRNAs in the adrenal glands of Sunite sheep (Ovis aries)
Source: Front Vet Sci. 2022 Jul 22;9:888207. doi: 10.3389/fvets.2022.888207 (PMC9354845; doi:10.3389/fvets.2022.888207)
Supplement: Supplementary file 1 [file Data_Sheet_1.ZIP › Supplementary file/miRDeep2 file.zip!/miRDeep2 file/Know_Structure/LP42A2/oar-mir-218a.pdf]

miRBase precursor : oar-mir-218a
Total read count : 56590
oar-miR-218a read count : 56589
remaining reads : 1

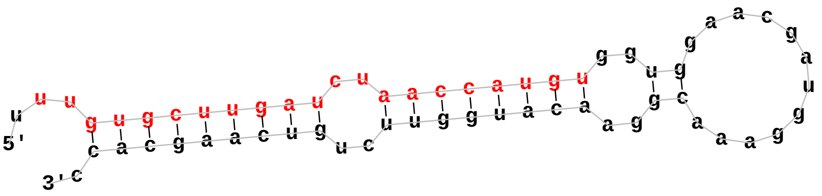

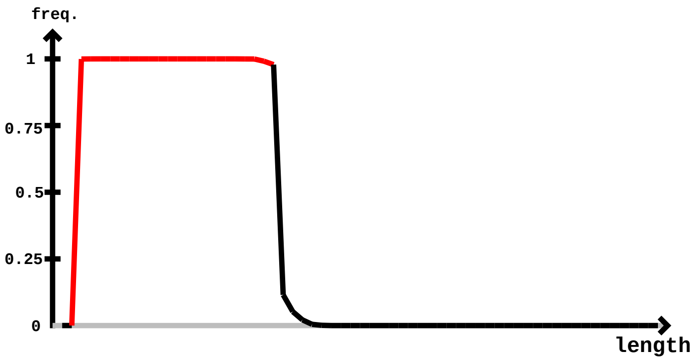


```
     oar-miR-218a
5'- uuugugcuugaucuaaccaugugguggaacgauggaaacggaacaugguucugucaagcacc   -3'    exp
    ...(((((((((..((((((((..((............))..))))))))..))))))))).   reads      mm         sample
    Guugugcuugaucuaaccaugu........................................   1          1          seq
    uuugugcuugaucuaaccaugu........................................   2          0          seq
    uuugugcuugaucuaaccauguggu.....................................   1          0          seq
    .uugugcuugaucuaacA............................................   1          1          seq
    .uugugcuugaucuaacc............................................   12         0          seq
    .uugAgcuugaucuaacc............................................   1          1          seq
    .uugugcuugaucuaacca...........................................   14         0          seq
    .uugugcuugaucuaacAau..........................................   1          1          seq
    .uugugcuugaucuaaccau..........................................   470        0          seq
    .uugugcuugaucuaaccaug.........................................   712        0          seq
    .uugugcuugaucuaacAaug.........................................   2          1          seq
    .uugugcuugaucuaaccaugu........................................   48688      0          seq
    .uugugcuugaucuaacAaugu........................................   155        1          seq
    .uugugcuugaucuaaccaugug.......................................   3563       0          seq
    .uugugcuugaucuaaccaugugg......................................   1731       0          seq
    .uugugcuugaucuaaccauguggu.....................................   941        0          seq
    .uugugcuugaucuaaccaugugguU....................................   200        1          seq
    .uugugcuugaucuaaccauguggug....................................   11         0          seq
    .uugugcuugaucuaaccaugugguUg...................................   53         1          seq
    .uugugcuugaucuaaccauguggugg...................................   1          0          seq
    ..ugugcuugaucuaaccaugu........................................   6          0          seq
    ..ugugcuugaucuaaccaugugg......................................   13         0          seq
    ..ugugcuugaucuaaccauguggu.....................................   2          0          seq
    ..ugugcuugaucuaaccaugugguU....................................   3          1          seq
    ...gugcuugaucuaaccaugu........................................   4          0          seq
    ...gugcuugaucuaaccaugug.......................................   1          0          seq
    ...........................................caugguucCgucaagcacc   1          1          seq
```
